# Single-Cell Spatial Analysis of Tumor and Immune Microenvironment on Whole-Slide Image Reveals Hepatocellular Carcinoma Subtypes

**DOI:** 10.3390/cancers12123562

**Published:** 2020-11-28

**Authors:** Haiyue Wang, Yuming Jiang, Bailiang Li, Yi Cui, Dengwang Li, Ruijiang Li

**Affiliations:** 1 Shandong Key Laboratory of Medical Physics and Image Processing, Shandong Institute of Industrial Technology for Health Sciences and Precision Medicine, School of Physics and Electronics, Shandong Normal University, Shandong 250358, Jinan, China; Haiyue23@stanford.edu; 2 Department of Radiation Oncology, Stanford University School of Medicine, Palo Alto, CA 94304, USA; ymjiang2@stanford.edu (Y.J.); bli2@stanford.edu (B.L.); cuiyi@stanford.edu (Y.C.)

**Keywords:** spatial analysis, histopathology image, deep learning, tumor microenvironment, hepatocellular carcinoma

## Abstract

**Simple Summary:**

Current molecular classification systems are primarily based on cancer-cell-intrinsic features, which disregard the critical contribution of the microenvironment and lack spatial information. Here, we take a holistic approach by incorporating spatial imaging phenotypes of both tumor and immune microenvironment for integrated classification. To achieve this goal, we developed a deep learning pipeline for automated nuclei segmentation and classification at the single-cell level. By leveraging this tool, we performed quantitative phenotypic characterization of tumor cells and infiltrating lymphocytes as well as their spatial distribution and relation. Using whole-slide hematoxylin- and eosin-stained images of hepatocellular carcinoma, we identified three histological imaging subtypes that are associated with distinct molecular features and clinical outcomes. This study represents an important step forward in understanding the spatial heterogeneity and complex interactions between tumor and immune microenvironment as well as their clinical implications.

**Abstract:**

Hepatocellular carcinoma (HCC) is a heterogeneous disease with diverse characteristics and outcomes. Here, we aim to develop a histological classification for HCC by integrating computational imaging features of the tumor and its microenvironment. We first trained a multitask deep-learning neural network for automated single-cell segmentation and classification on hematoxylin- and eosin-stained tissue sections. After confirming the accuracy in a testing set, we applied the model to whole-slide images of 304 tumors in the Cancer Genome Atlas. Given the single-cell map, we calculated 246 quantitative image features to characterize individual nuclei as well as spatial relations between tumor cells and infiltrating lymphocytes. Unsupervised consensus clustering revealed three reproducible histological subtypes, which exhibit distinct nuclear features as well as spatial distribution and relation between tumor cells and lymphocytes. These histological subtypes were associated with somatic genomic alterations (i.e., aneuploidy) and specific molecular pathways, including cell cycle progression and oxidative phosphorylation. Importantly, these histological subtypes complement established molecular classification and demonstrate independent prognostic value beyond conventional clinicopathologic factors. Our study represents a step forward in quantifying the spatial distribution and complex interaction between tumor and immune microenvironment. The clinical relevance of the imaging subtypes for predicting prognosis and therapy response warrants further validation.

## 1. Introduction

Hepatocellular carcinoma (HCC) represents the majority of liver cancer, which is one of the most prevalent malignancies and a leading cause of cancer-related deaths worldwide [1]. HCC is known to be a heterogeneous disease with diverse characteristics and clinical outcomes [2,3,4]. Various classification systems have been proposed to divide patients into different groups or subtypes, most of which are defined according to tumor-intrinsic histopathologic or molecular features [5,6,7,8,9,10,11]. It has been increasingly recognized that in addition to neoplastic cells, the tumor microenvironment (TME) also plays an important role in cancer progression, metastasis, therapeutic response, and resistance [12,13]. In particular, several immune-microenvironment-based molecular classifications have recently been proposed for HCC [14,15,16].

Molecular classification requires analysis of high-throughput sequencing data measured in a small piece of tissue, making it susceptible to sampling bias as HCC has been shown to exhibit intratumor heterogeneity [17,18]. Another limitation with molecular analysis is that information about the location and spatial distribution of various cell types is lost. On the other hand, histology image-based analysis allows direct visualization of the cellular phenotypes in situ, providing a unique perspective into the tumor and the immune microenvironment [19,20,21]. While pathologist assessment remains the gold standard, this manual approach is limited by intra/interobserver variability and is unable to fully process the vast amount of information contained in the image.

Deep learning has recently emerged as a powerful technique for pathologic image analysis [22,23]. Promising performance has been reported in a variety of clinical applications, such as detecting breast cancer lymph node metastases [24], diagnosing prostate cancer grade [25], predicting lung cancer mutations [26], and predicting patient outcomes [27,28]. However, the vast majority of studies have focused on image analysis at the patch level, which may contain a mixture of up to several dozen cells. This patch-based approach cannot provide detailed information at the single-cell resolution.

In this study, we take a holistic approach by incorporating both tumor- and immune microenvironment-related features for the integrated classification of HCC. To achieve this goal, we developed a deep-learning-based pipeline for automated single-cell segmentation and classification on whole-slide images of tissue sections. By leveraging this tool, we performed quantitative phenotypic characterization of tumor cells and infiltrating lymphocytes as well as their spatial distribution and relation. We identified novel histological subtypes of HCC that are associated with distinct prognoses and molecular features.

## 2. Results

### 2.1. Automated Nuclei Detection and Cell Type Identification by Deep Learning

We developed a deep-learning-based pipeline to automatically segment and classify individual nuclei on whole-slide hematoxylin- and eosin-stained (H&E) images (Figure S1). Here, we collected publicly available images for 304 patients in the Cancer Genome Atlas Hepatocellular Carcinoma (TCGA–LIHC) cohort. For training and testing purposes, we manually segmented more than 65,000 nuclei in 1800 image patches from 66 randomly selected patients and labeled each nucleus as one of the following three cell types: tumor cells, lymphocytes, and other nonmalignant cells. 

**Figure 1:**
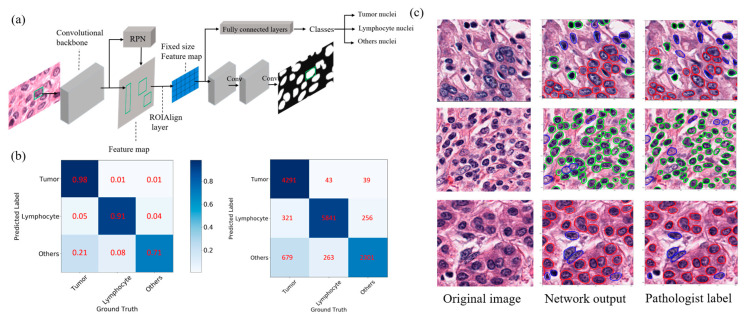
Proposed deep learning scheme for automated nuclei segmentation and classification based on Mask R-CNN (**a**). The confusion matrices for cell-type classification are shown for the testing dataset (**b**). Values are the percentage and number of nuclei correctly (diagonal) and incorrectly (off-diagonal) classified by the algorithm. Visual representation of nuclei segmentation and classification for some examples are shown in (**c**). The magnification for all images is 40×. Red, green, blue outlines for individual nuclei correspond to those of tumor cells, lymphocytes, and other cells, respectively. RPN: region proposal network.

In both training and testing datasets, the proposed deep learning pipeline based on Mask region-based convolutional neural network (R-CNN) achieved a high accuracy of nuclei detection at 92%, with precision and recall rates of 97% and 94%. For identification of cell types, the deep learning model was highly accurate in the training dataset, with an overall classification of 99% for tumor cells and 97% for lymphocytes, respectively. Similarly, high classification accuracies were also observed in the testing dataset: 98% for tumor cells and 91% for lymphocytes, as shown in Figure 1b. The classification for other cells was moderately accurate (71–81%). Some representative images in the testing set are shown in Figure 1c, and additional images without pathologist labels are shown in Figure S2, which confirm the accurate segmentation and classification of the deep learning model.

### 2.2. Discovery and Validation of Imaging Subtypes

**Figure 2:**
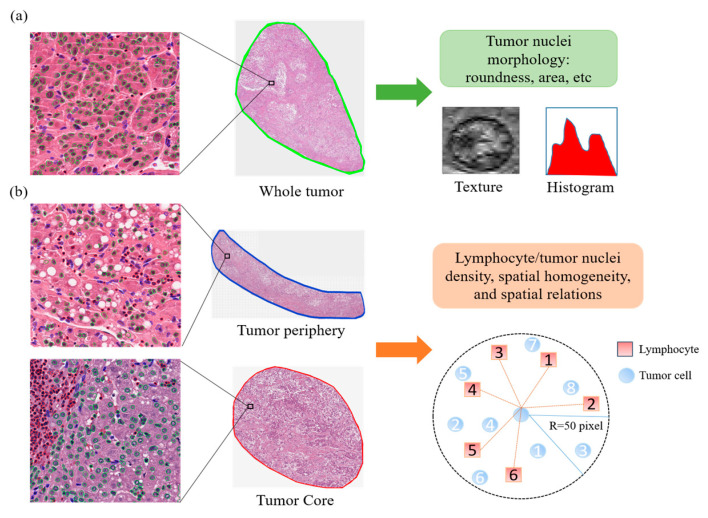
Feature extraction of pathological images. (**a**) For the whole tumor area, morphology, histogram, and texture features of the tumor nuclei are calculated. (**b**) For tumor core and tumor periphery, several types of features, including the density, spatial distribution, and relation between tumor cells and lymphocytes, are calculated. In particular, we evaluate the statistical features of the number of lymphocytes surrounding each tumor cell. Red, green, blue outlines for individual nuclei correspond to those of tumor cells, lymphocytes, and other cells, respectively. The magnification for all images is 40×.

**Figure 3:**
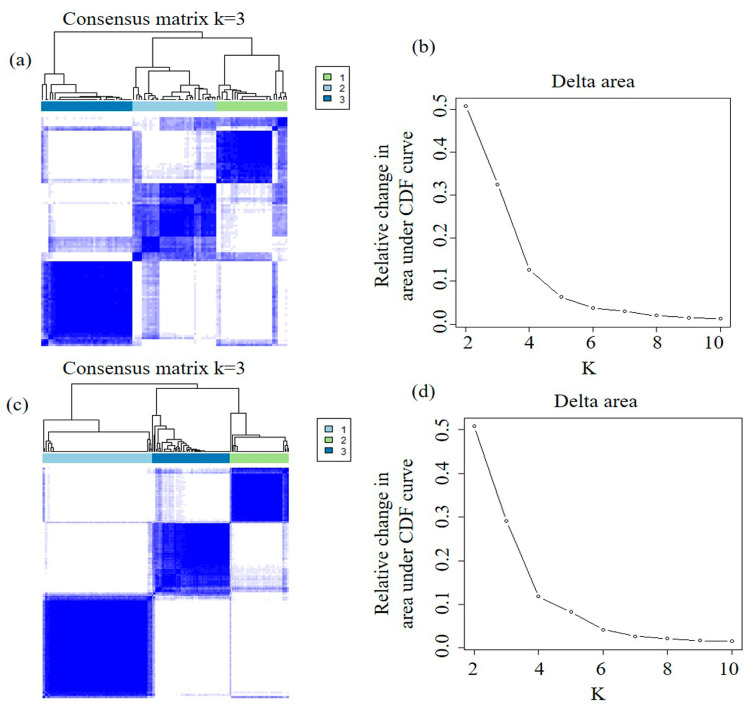
Discovery and validation of the imaging subtypes. (**a**,**c**) represent the consensus matrices of the heatmaps in the discovery and validation cohorts, respectively. Patient samples are both rows and columns, and consensus values range from 0 (never clustered together) to 1 (always clustered together). The optimal cluster number (K = 3) is determined by the area under the cumulative distribution function (CDF) curve (**b**,**d**), which corresponds to the largest number of clusters that induced the smallest incremental change in the area under the CDF curves.

Given the detailed single-cell map obtained earlier, we calculated 246 quantitative image features to characterize individual nuclei as well as spatial distribution and relation between tumor cells and lymphocytes in three different regions, including whole tumor, tumor core, and tumor periphery (Figure 2). Based on these image features, we applied unsupervised consensus clustering to identify imaging subtypes in the TCGA discovery set. The optimal number of clusters was determined to be 3, which maximized consensus within clusters and minimized the rate of ambiguity in cluster assignments, as shown in Figure 3. 

To assess the reproducibility of the imaging subtypes, we independently applied the same clustering algorithm in the TCGA validation set. This analysis confirmed the optimal number of clusters as 3. Further, we computed the in-group proportion (IGP) statistic to quantify the similarity of the imaging subtypes between the discovery and validation sets. Imaging Subtypes 1 and 2 showed a high consistency between the 2 cohorts, with the corresponding IGP values at 80% and 90%, respectively. Imaging Subtype 3 was associated with a lower IGP of 67% but remained statistically significant (*p* < 0.001).

### 2.3. Tumor and Immune Microenvironment Features of Imaging Subtypes

**Figure 4:**
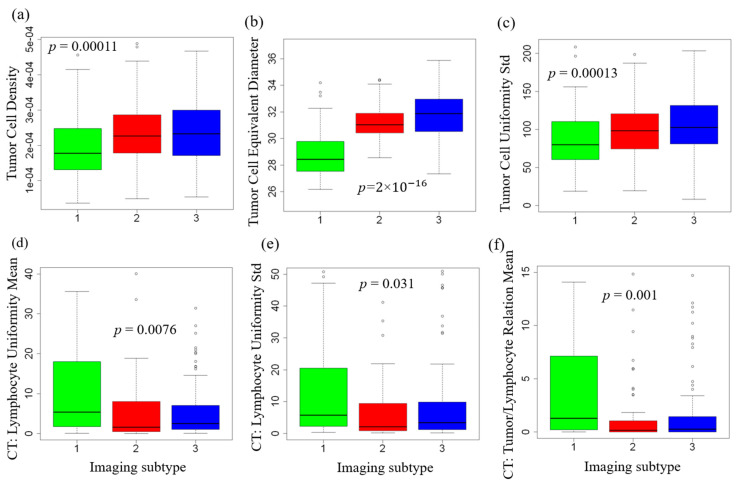
Selected quantitative imaging features that are significantly associated with the three imaging subtypes. All *p*-values are corrected for multiple testing. CT: tumor core. (**a**) tumor nuclei density; (**b**) tumor nuclei equivalent diameter; (**c**) tumor nuclei uniformity standard deviation; (**d**) lymphocyte uniformity mean; (**e**) lymphocyte uniformity standard deviation; (**f**) tumor and lymphocyte relation mean. These features are explained in Supplemental Table S1.

To better understand specifically which imaging features distinguish these subtypes, we performed statistical analysis with multiple testing correction and found many features were significantly associated with the imaging subtypes. Some representative examples are shown in Figure 4. Specifically, Imaging Subtype 1 was characterized by the lowest density of tumor cells and the least heterogeneity in terms of spatial distribution among all three subtypes. In comparison, Imaging Subtypes 2 and 3 were associated with a higher density of tumor cells with heterogeneous distributions (Figure 4a,c), which is likely due to increased tumor cell growth and proliferation. Subtypes 2 and 3 were further characterized by larger tumor nuclei (Figure 4b) that may be associated with polyploidy. 

Regarding the spatial distribution of tumor-infiltrating lymphocytes (TIL), intratumoral TIL density was highest for Subtype 1 and much lower for Subtypes 2 and 3 (Figure 4d). The same is true for the spatial heterogeneity of TIL distribution (Figure 4e). Importantly, we observed that the average density of TIL surrounding each tumor cell was highest for Subtype 1 and significantly lower for Subtypes 2 and 3 (Figure 4f). This suggests that Subtype 1 is enriched for tumors in which the infiltrating lymphocytes and tumor cells tend to be spatially clustered or colocalized. Similar patterns regarding the spatial distribution of lymphocytes and the relation with tumor cells were observed in the tumor periphery (Figure S3). 

It is worth noting that although tumors with each subtype are enriched for the corresponding characteristics, this is by no means a strict rule. For instance, not all tumors with high lymphocyte infiltration were classified as Subtype 1, and vice versa. This is because the tumor cell density also had to be low for Subtype 1, and these two features were only weakly correlated (Pearson correlation: −0.17). Therefore, our subtyping system incorporates complementary information from both tumor- and immune-related features. 

### 2.4. Molecular Pathways Associated with Imaging Subtypes

**Figure 5:**
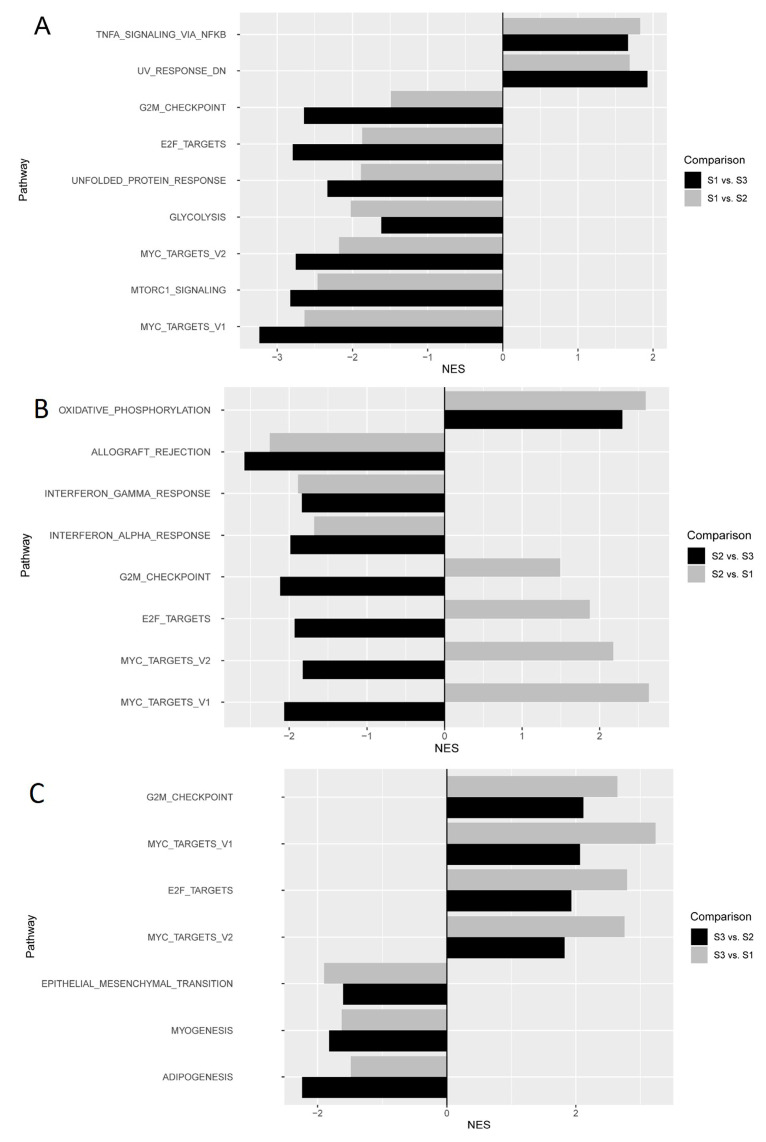
Molecular pathways significantly enriched for each of the three imaging subtypes via pairwise comparison. Cancer hallmark pathways were used for the gene set enrichment analysis. (**A**) S1 vs. S2 and S3; (**B**) S2 vs. S1 and S3; (**C**) S3 vs. S1 and S2.

To investigate the underlying molecular characteristics for individual histological subtypes, we performed differential gene expression analysis by pairwise comparison and then conducted GSEA analysis on the ranking list of differentially expressed genes (Figure 5). We found that Imaging Subtype 1 was associated with significantly upregulated TNFα signaling, which suggests increased inflammation and immune infiltration. In addition, Subtype 1 had the lowest level of mTOR signaling, E2F, and MYC targets, which are related to cell cycle progression, cell growth, and proliferation. By contrast, Subtype 2 was associated with the lowest level of INFγ and INFα signaling among all three subtypes, which indicates the absence of an effective antitumor immune response. Interestingly, Subtype 2 had the highest level of oxidative phosphorylation. For Subtype 3, the most prominent feature was the increased expression of genes related to cell cycle progression and proliferation, which is the highest among the three subtypes. These observations are largely consistent with the histological characteristics of the imaging subtypes.

### 2.5. Relation to Established Molecular Subtypes and Genetic Alterations

We compared our histologic imaging subtypes with established molecular classification based on either tumor-intrinsic genomic or immune-related features (Figure S4). We found that TCGA integrative genomic clusters (iClusters) were almost uniformly distributed among the imaging subtypes (*p* = 0.762), suggesting that the two classification systems are independent of each other. Similarly, the pan-cancer immune subtypes in the TCGA–LIHC cohort were also unrelated to the proposed imaging subtypes (*p* = 0.294), with a near-uniform distribution. There was no association between the imaging subtypes and major driver genetic alterations in *TP53* or *CTNNB1* in HCC (Figure S5). However, we did observe a significant association (*p* = 0.018) between imaging subtypes and aneuploidy (Figure S6), which is consistent with the findings on tumor nuclei size (Figure 5B).

### 2.6. Prognostic Impact of Imaging Features and Subtypes

**Figure 6:**
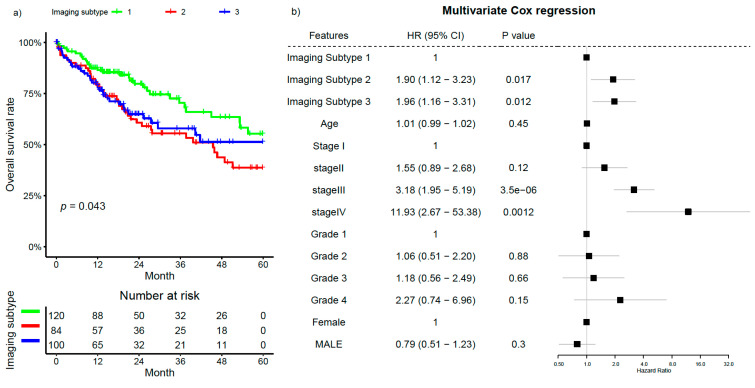
Kaplan–Meier curves for overall survival of patients stratified by the imaging subtypes (**a**), and forest plot for the multivariate Cox regression analysis (**b**) in the TCGA cohort. Imaging Subtype 1, Stage I, Grade 1, and female were used as the baseline in multivariate analysis.

We evaluated the prognostic impact of the imaging subtypes in the TCGA cohort. Overall, the patients with Subtype 1 had the best prognosis among the three subtypes, while those with Subtypes 2 and 3 had a worse prognosis (Figure 6a). There were no differences in survival between Subtypes 2 and 3. When adjusting for other clinicopathologic factors, including tumor stage and grade, Imaging Subtypes 2 and 3 were still associated with a statistically significant worse prognosis compared to Subtype 1 in multivariable analysis (Figure 6b). Finally, given the known correlation with imaging subtypes, we tested whether a simple feature of tumor/lymphocyte spatial relation (i.e., the average number of lymphocytes per tumor cell) could be used to stratify patients. Indeed, this image feature was again significantly associated with survival (HR: 0.56 [0.38–0.84], *p* = 0.0043, Figure S7).

## 3. Discussion

In this study, we performed quantitative, single-cell characterization of the histological imaging phenotypes, including spatial distribution and relations between tumor cells and the immune microenvironment. We identified three reproducible histological subtypes of HCC that are associated with distinct molecular features and prognoses. Further, these novel histological imaging subtypes complement established molecular classification and demonstrate independent prognostic value beyond conventional clinicopathologic risk factors in HCC. 

The proposed histologic subtyping is distinct from current molecular classification focusing on either cancer-cell-intrinsic or immune features. Rather, we take a holistic approach by incorporating spatial imaging phenotypes of tumors and immune microenvironments for integrated classification of HCC. Molecular profiling based on a needle biopsy suffers from inherent sampling bias due to intratumor heterogeneity. By contrast, in situ, image-based analysis has the unique advantage of providing the spatial context and looking at the entire tumor from a global perspective. Here, we developed a deep-learning-based pipeline for automated and accurate single-cell segmentation and classification, which transforms the whole-slide pathological image into a detailed “spatial map” of different cell types in the tumor. This allows a comprehensive analysis of the spatial distribution and relations among tumor cells and lymphocytes.

Our study is among the first to apply deep learning and computational approaches to systematically quantify the spatial imaging phenotypes of tumors and immune microenvironments at the single-cell level. To date, most studies have focused on the evaluation and prediction of known pathologic or genomic features of the tumor using a patch-based analysis [26,27,29,30]. For instance, Chen et al. trained a deep neural network on hematoxylin- and eosin-stained whole-slide images for benign and malignant classification, histologic grading, and predicting commonly mutated genes in HCC [31]. In another study, a deep-learning-based model was developed to differentiate between HCC and cholangiocarcinoma, which achieved an accuracy of 0.842 on an independent test set [32]. However, when the model’s prediction was incorrect, assistance with deep learning significantly decreased the accuracy of pathologists. Several recent studies have investigated the prognostic relevance of TILs in terms of density and spatial distribution in various cancer types. Of note, Saltz et al. used deep learning to classify TILs in a 50-micron image patch on whole-slide images (WSI) and performed a comprehensive analysis to define the spatial pattern of TILs in 13 cancer types [19]. While this pan-cancer study generated a large amount of data and is an invaluable resource, the patch-based analysis did not allow detailed characterization of tumor cells and TILs at the single-cell resolution. 

In our study, we revealed a subset of HCC that demonstrated a large number of neighboring lymphocytes per tumor cell, suggesting a spatial pattern of colocalization between the two cell types. Further, this group of patients had superior prognoses. Consistent with this finding, Yuan and colleagues proposed an immune spatial score based on cancer-immune hotspot analysis and demonstrated its prognostic significance in breast cancer [33,34]. In another recent study, Corredor et al. used handcrafted image features to identify TILs in a tissue microarray (TMA) and showed that spatial architecture of TILs was associated with the risk of recurrence in early-stage nonsmall cell lung cancer [35]. While evaluation of small TMA cores may suffer from potential sampling bias, whole-slide images used in our study are more representative of the tumor. Our work, along with these recent studies, highlights the importance of investigating spatial distribution beyond the density of TILs, which represent different concepts of the immune landscape. 

It is worth noting that the primary purpose of our study is not for specific diagnostic and prognostic applications. Rather, our goal is the discovery of histological patterns by incorporating spatial distributions and relations at the single-cell level in order to better understand the complex interplay between tumor and immune microenvironment. It remains to be determined how the single-cell analysis would compare with traditional patch-based approaches for the purpose of diagnosis and prediction of treatment response and outcomes. It is clear that these two approaches provide complementary information at different spatial scales and combining them might ultimately lead to better results than either one used alone.

By integrating histological imaging and molecular data, we showed that Imaging Subtype 1 was characterized by low proliferation and the presence of antitumor immune response signaling, which is consistent with their overall best prognosis. While Subtype 2 had an intermediate level of proliferation, it was also associated with an immune-excluded phenotype, which is unlikely to benefit from immune checkpoint inhibitors alone. The finding that Subtype 2 had increased oxidative phosphorylation is intriguing because inhibitors of oxidative phosphorylation could be used to therapeutically target this subtype to alleviate adverse tumor hypoxia [36]. Several drugs, including metformin, may potentially be used to target cancer cell metabolism in patients of Subtype 2. On the other hand, Subtype 3 exhibited high tumor cell growth and proliferation, which, in turn, was associated with a worse prognosis. This finding is consistent with many previous studies showing that proliferative HCC tends to be more aggressive and poorly differentiated and have adverse outcomes [10]. Novel therapeutic strategies targeting this group of patients are needed to improve their survival rate.

We found that the Imaging Subtypes 2 and 3 were associated with higher aneuploidy, which seems consistent with the larger tumor nuclei size and worse prognosis. However, we did not find any association between the imaging subtypes and key genetic alterations in *TP53* or *CTNNB1* in HCC. This might be because our imaging subtypes were discovered via unsupervised clustering and were mainly driven by features related to the spatial distribution of tumor cells and their relations to lymphocytes. While it is not the purpose of our study, it is possible that a supervised approach focusing on tumor nuclei features may lead to useful predictions of genetic mutations.

There are several limitations to this study. First, our analysis is focused on tumor cells and lymphocytes, while other immune and stromal cells were grouped into the third category. Second, given the inherent limitation of H&E-stained images, we cannot distinguish subsets of lymphocytes such as cytotoxic and memory T-cells or B-cells, which may play different roles in adaptive immune response [37]. To better distinguish these heterogeneous cell populations, more comprehensive labeling tools such as multiplex immunohistochemistry staining will be required [38]. In principle, our computational pipeline will also be applicable to these situations with some modification. Another caveat is that the molecular data obtained by bulk tumor profiling may be confounded by spatial heterogeneity, which could affect our analysis for association with histopathologic data.

## 4. Methods

### 4.1. Study Design

In this study, we first trained a deep neural network on whole-slide images to automatically segment and classify individual nuclei into three different cell types. Second, we extracted quantitative imaging features of the tumor and the immune microenvironment as well as their spatial distribution and relation. Third, we discovered novel imaging subtypes based on unsupervised clustering and independently evaluated the reproducibility. Finally, we investigated the prognostic relevance and molecular features associated with the imaging subtypes. 

### 4.2. Patients and Datasets

Publicly available whole-slide hematoxylin- and eosin-stained (H&E) images for the Cancer Genome Atlas Hepatocellular Carcinoma (TCGA-LIHC) cohort were retrieved from the cancer imaging archive (www.cancerimagingarchive.net). After excluding 73 cases due to poor image quality, 304 cases were included in this study. For identifying imaging subtypes, we divided the TCGA–LIHC cohort with a ratio of approximately 1:2 into a discovery set (*n* = 99) and a validation set (*n* = 205) using a computer-based random number generator. Patient characteristics are summarized in Table 1.

### 4.3. Automated Nuclei Segmentation and Cell Type Identification

We trained a deep neural network to segment and classify individual nuclei into three cell types: tumor cells, lymphocytes, and other nonmalignant cells. The Mask R-CNN model is a widely used multitask deep learning neural network architecture for object detection and segmentation and has demonstrated superior performance in benchmark experiments [39]. The architecture of Mask R-CNN consists of a convolutional neural network as the backbone for feature extraction and a network head for object recognition and class prediction (Figure 1a). Here, we adapted the Mask R-CNN model for our nuclei segmentation and classification tasks. To train the network, we manually segmented and labeled more than 65,000 nuclei in 1700 image patches that were randomly selected from 66 cases. Details of network training are in Supplemental Methods. The cell annotation datasets and software code are publicly available and can be accessed via the following link: https://github.com/zilanjiuwan/Single-Cell-Imaging-Analysis-of-HCC-Data.git.

### 4.4. Quantitative Image Feature Extraction

To obtain more complete information about the tumor, we analyzed three regions of interest (ROI) on the whole-slide image (WSI): whole tumor, tumor core, and tumor periphery (Figure 2). The whole tumor is outlined to include the extent of gross disease; the tumor periphery is defined by the outermost part of the tumor; the tumor core is a region inside the tumor (mostly consisting of tumor cells, excluding intratumoral stroma) not extending to the tumor periphery. Each ROI was manually delineated using the annotation tool of Image Scope software by a pathologist (Y.J.) with 6 years of experience. Within each ROI, we applied the deep learning model to convert the pathological image into a spatial map of tumor cells, lymphocytes, and other nonmalignant cells. From this map, we can define specific image features of individual nuclei and further characterize the spatial relation between tumor cells and lymphocytes. 

In detail, we extracted a total of 246 quantitative image features (Table S1). Specifically, we performed feature calculations on individual tumor nuclei in the whole tumor region. The tumor-nuclei-related features included 20 morphological features describing the geometry and size of tumor nuclei, 14 image histogram intensity features describing the first-order statistical properties, 62 texture features, and 54 color features. In the tumor core and the tumor periphery, we calculated 28 features related to the density and homogeneity of the tumor nuclei and the lymphocyte nuclei in terms of their spatial distribution. To further characterize the spatial interaction between tumor and immune cells, we extracted 64 image features from both regions. To calculate the spatial interaction matrix, each tumor nucleus was expanded by a radius of 50 or 100 pixels and the number of tumor and lymphocyte nuclei present in this range was recorded, respectively, as shown in Figure 3b. A variety of statistical properties of the spatial interaction matrix were computed. Image analysis and feature extraction were implemented with OpenCV software in Python.

### 4.5. Imaging Subtype Discovery and Validation

To dissect intertumor heterogeneity defined by histologic phenotypes, we applied unsupervised consensus clustering [40] to define a subgroup of patients, i.e., imaging subtypes, that demonstrate distinct single-cell image features, as described above. Specifically, we used the partition-around-medoid clustering algorithm with the Spearman distance metric and performed 10,000 bootstraps, with 80% resampling of the image features. The consensus clustering algorithm was implemented in the ConsensusClusterPlus software package. The optimal number of clusters was determined by the most stable consensus matrix and explicit cluster allocation across permuted runs. To assess the reproducibility of the clusters, we performed the same clustering procedure independently in the validation set to identify image subtypes. We computed the in-group proportion (IGP) statistic to quantitatively measure the similarity of clusters defined in the two datasets.

### 4.6. Functional Enrichment Analyses for Imaging Subtypes

We conducted Gene Set Enrichment Analysis (GSEA) [41] for the histologic imaging subtypes using the TCGA–LIHC cohort. Normalized RNA-seq data were retrieved from the Genomic Data Commons. We first estimated the mean expression level of a certain gene in different imaging subtypes via a linear model. Then, the difference in the average gene expression level between any two imaging subtypes was estimated with an empirical Bayes version of the moderated *t*-test [42]. We conducted three pairwise comparisons among the three subtypes. For the enrichment analysis, we focused on the cancer hallmark gene sets from the molecular signature database or MSigDB [43] and only reported enriched gene sets with a false discovery rate (FDR) < 0.01.

### 4.7. Relation between Imaging Subtypes and Established Genetic and Molecular Subtypes

We compared our histological imaging subtypes with two existing molecular classification systems: TCGA PanCancer immune subtypes [44] and TCGA integrative genomic clusters (iCluster) for HCC [9]. Briefly, the pan-cancer immune subtypes divided the cancer patients into six subgroups (C1–C6), reflecting distinct tumor immune status, and the patients in the TCGA–LIHC dataset were mostly classified into C1 to C4 subgroups. By integrating large-scale multiplatform data, the TCGA consortium stratified patients into three different integrative clusters (iCluster 1–3). The relation between imaging subtypes and aneuploidy, as well as mutations in *TP53* and *CTNNB1*, two major driver genetic alterations in HCC, were also investigated.

### 4.8. Statistical Analysis

For comparisons of more than two groups, one-way analysis of variance (ANOVA) and Kruskal–Wallis tests were used as parametric and nonparametric methods, respectively. We used the one-way ANOVA test to identify quantitative image features that were significantly associated with the imaging subtypes. Multiple statistical testing was corrected by the Benjamini–Hochberg method. Kaplan–Meier analysis and a log-rank test were used to evaluate patient stratification into different risk groups. The relationship between imaging and molecular subtypes was assessed with a chi-squared test. All statistical tests were two-sided, and *p*-values of less than 0.05 were considered statistically significant. Statistical analyses were conducted using R (version 3.6.1). 

## 5. Conclusions

We discovered three histological subtypes of HCC by single-cell characterization of the quantitative imaging phenotypes. Our study represents a step forward in quantifying the spatial distribution and complex interaction between tumors and their immune microenvironments. The relevance of the imaging subtypes for predicting prognosis and therapy response warrants further validation in diverse clinical settings.

## Figures and Tables

**Table 1:** Clinicopathologic characteristics of patients in the discovery and validation cohorts.

Variables	Discovery Cohort (*n* = 99)		Validation Cohort (*n* = 205)		*p* Value *
	N	%	N	%	
**Gender**					0.44
Female	37	37%	66	32%	
Male	62	63%	139	68%	
**Age (years)** **Median (Interquartile Range)**	60 (50–68)		60 (51–69)		0.72
**Primary tumor stage**					<0.01
pT1	29	29%	115	56%	
pT2	28	28%	53	26%	
pT3	36	36%	32	16%	
pT4	6	6%	3	1%	
Unknown	0	0%	2	1%	
**Grade**					0.15
G1	16	16%	26	13%	
G2	50	51%	92	45%	
G3	32	32%	73	36%	
G4	0	0%	11	5%	
Unknown	1	1%	3	1%	

* The differences in categorical variables were tested using the chi-squared test. The difference in age was tested using the Wilcoxson test.

## Supplementary Materials

The following are available online at https://www.mdpi.com/2072-6694/12/12/3562/s1. Figure S1: Flowchart of the image processing pipeline; Figure S2: Visual representation of nuclei segmentation and classification for some examples without pathologist labels; Figure S3: Selected quantitative imaging features that are significantly associated with the three imaging subtypes in the tumor periphery; Figure S4: Relation between the proposed histological imaging subtypes and established molecular subtypes; Figure S5: Relation between the histological imaging subtypes and genetic mutations in HCC; Figure S6: Relation between the histological imaging subtypes and aneuploidy in HCC; Figure S7: Kaplan–Meier curves for overall survival of patients stratified by the median average number of lymphocytes per tumor cell; Table S1: Details of 246 quantitative image features extracted from whole-slide images.
