# Supplementary material for: Single-Cell Spatial Analysis of Tumor and Immune Microenvironment on Whole-Slide Image Reveals Hepatocellular Carcinoma Subtypes
Source: Cancers (Basel). 2020 Nov 28;12(12):3562. doi: 10.3390/cancers12123562 (PMC7761227; doi:10.3390/cancers12123562)
Supplement: Supplementary file 1 [file cancers-12-03562-s001.pdf]

Supplemental Materials

Single-Cell Spatial Analysis of Tumor and Immune Microenvironment on Whole-Slide Image Reveals Hepatocellular Carcinoma Subtypes

Haiyue Wang, Yuming Jiang, Bailiang Li, Yi Cui, Dengwang Li and Ruijiang Li

Supplemental Methods

Training of Deep Learning Network for Nuclei Segmentation and Classification

In order to properly train the Mask R-CNN model, we manually segmented all nuclei and labeled each as one of three cell types on 66 cases in the TCGA dataset. The Image Scope software was used to segment and label the nuclei by a pathologist (Y.J.) with 6 years of experience. Given the large size, we annotated a total of 1702 nonoverlapping image patches each with a size of 256 × 256, which were randomly assigned into a training set (80%) and testing set (20%). The training set contains 1358 image patches, with a total of 17,582 tumor cells, 22,550 lymphocytes, and 10,675 other non-malignant cells. The testing set contains 344 image patches, with a total of 4689 tumor cells, 7249 lymphocytes, and 3569 other non-malignant cells.

In this work, we employed ResNet-101 as the feature extraction network for Mask R-CNN model. Rather than training the network from scratch, we initialized the model using weights obtained from pre-training on the Microsoft COCO dataset [1]. To mitigate the effects of different H&E staining protocols, images were standardized (centered and scaled to have zero mean and unit variance) for each RGB channel. We performed extensive augmentations on the image patches in the training data by applying the following transformations: horizontal/vertical flipping, scaling, rotation, contrast normalization, affine transformation, and Gaussian blurring. We trained the network for 20,000 iterations, starting from a learning rate of 0.001, and reducing to 0.0002 at 16,000 and 0.0001 at 18,000 iterations. We used a weight decay of 0.0001 and a momentum of 0.9. The proposed network was implemented on the open source TensorFlow and Keras platform and trained using the NVIDIA Quadro P6000 GPU workstation.

Supplemental Figures

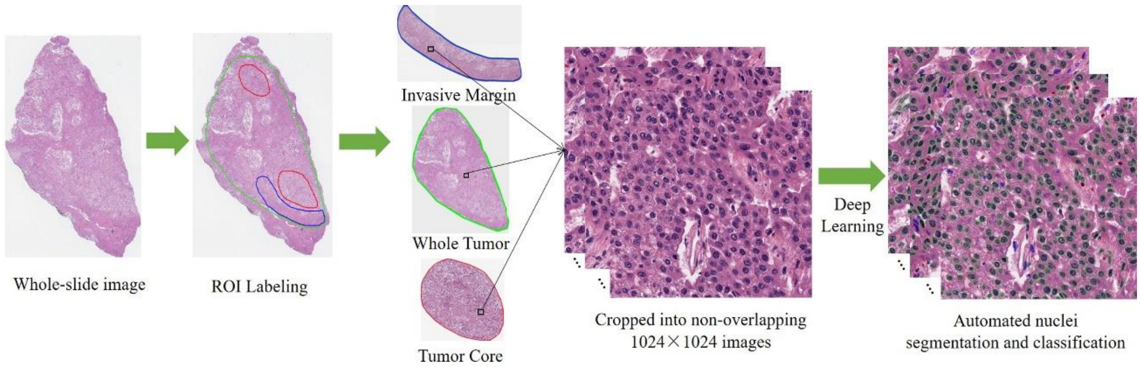

Figure S1. Flowchart of the image processing pipeline. For each whole-slide image, three different regions of interest (ROI) were manually delineated by a pathologist: whole tumor, tumor core, and invasive margin. A deep learning network was developed to perform automated nuclei segmentation and classification on all three ROIs. The magnification for all images is 40×.

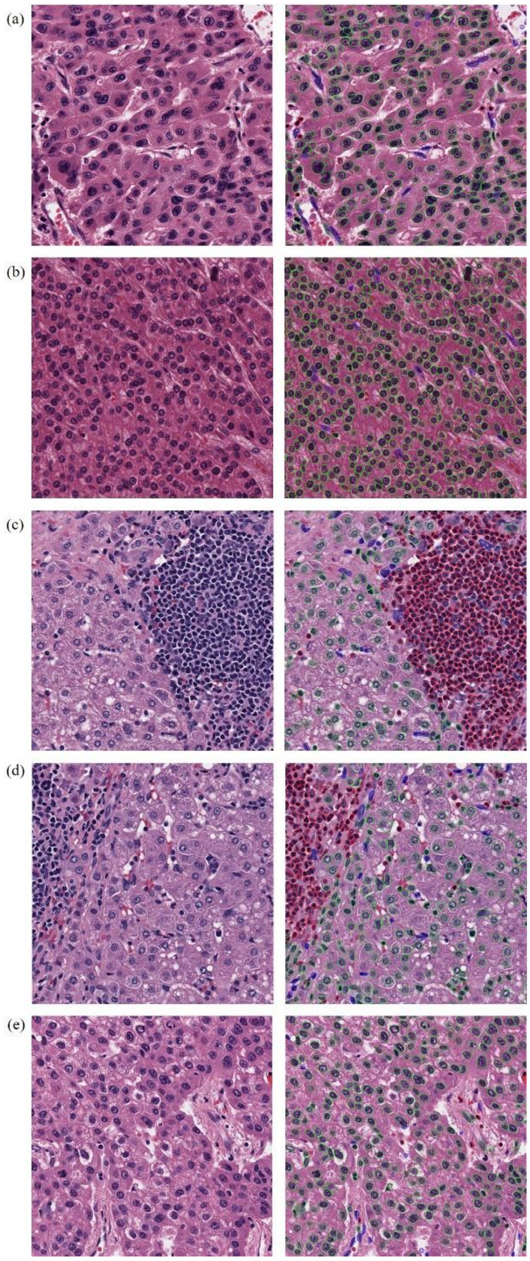

Figure S2. Visual representation of nuclei segmentation and classification for some examples without pathologist labels. The magnification for all images is 40×.

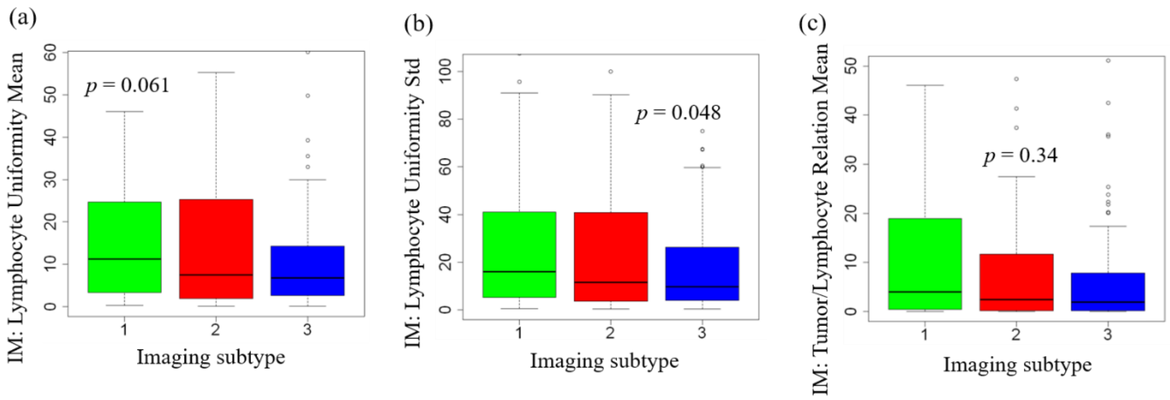

Figure S3. Selected quantitative imaging features that are significantly associated with the 3 imaging subtypes in the tumor boundary. (**a**) lymphocyte uniformity mean; (**b**) lymphocyte uniformity standard deviation; (**c**) tumor and lymphocyte relation mean.

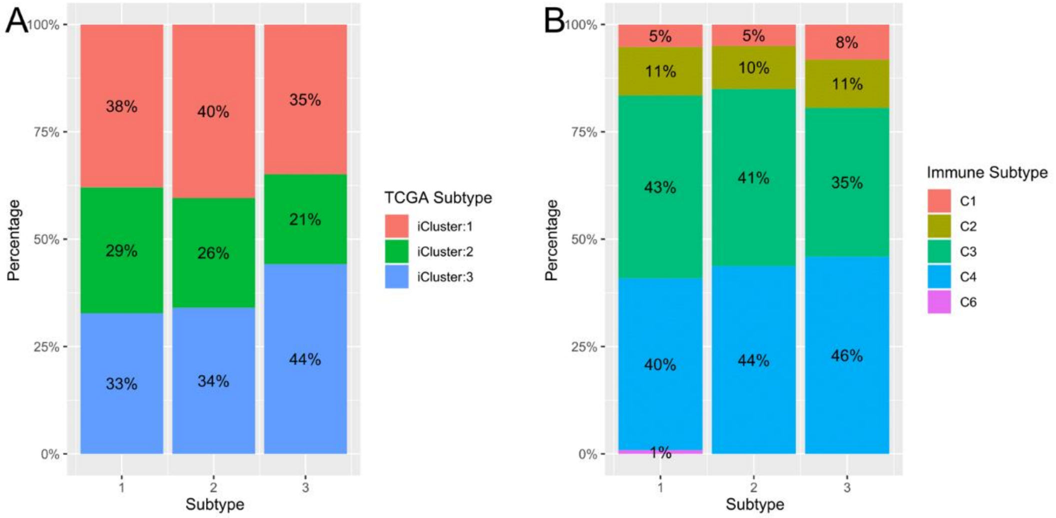

Figure S4. Relation between the proposed histological imaging subtypes and established molecular subtypes: (**A**) TCGA iClusters; (**B**) Pan-cancer immune subtypes.

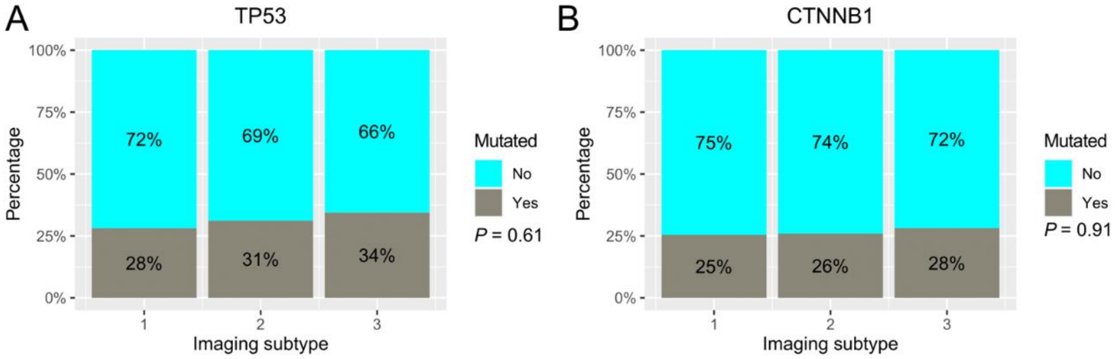

Figure S5. Relation between the histological imaging subtypes and genetic mutations in HCC. (**A**) distribution of TP53 mutation; (**B**) distribution of CTNNB1 mutation.

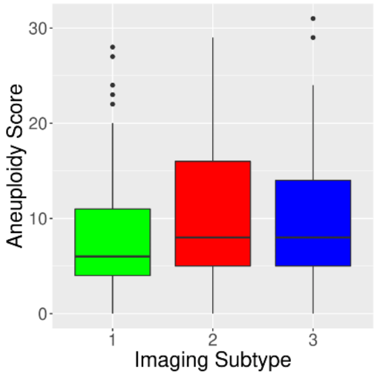

Figure S6. Relation between the histological imaging subtypes and aneuploidy in HCC (Kruskal-Wallis test $p = 0.018$).

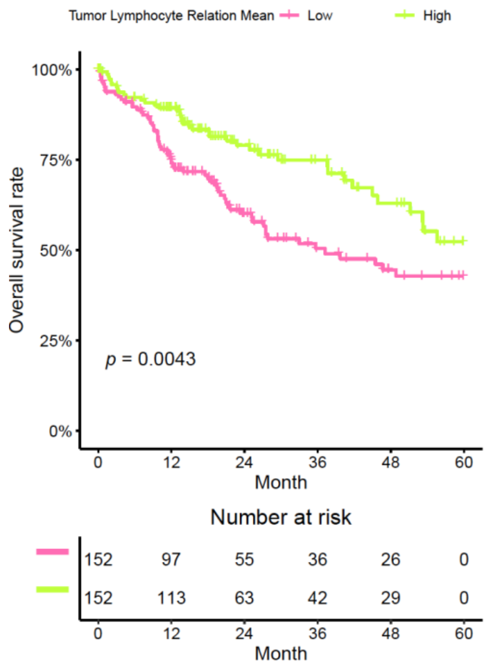

Figure S7. Kaplan–Meier curves for overall survival of patients stratified by the median average number of lymphocytes per tumor cell.

Supplemental Table

Table S1. Details of 246 quantitative image features extracted from whole-slide image.

ROI	Type	No.	Formulation	Interpretation
Whole Tumor	Morphology	20	Perimeter, Area, Radius of minimum enclosing circle, Circle center of minimum enclosing circle, Bounding rectangle area, Aspect Ratio, Extent, Rectangularity, Hull Area, Solidity, Equivalent Diameter, MA, ma, Angle, Fit Ellipse Area, Perimeter Area Ratio, Roundness, Compactness, Eccentricity.	Measure tumor nuclear shape, size, and boundary smoothness.
	Texture	62	***GLCM*** (Grey-Level Co-occurrence Matrix): Uniformity, Entropy, contrast, dissimilarity, homogeneity, correlation, ASM; ***GLRLM***(Gray Level Run Length Matrix-based feature): Short Run Emphasis(SRE), Long Run Emphasis(LRE), Gray Level Non-Uniformity(GLN), Run Length Non-Uniformity(RLN),Run Percentage(RP), Low Gray Level Run Emphasis (LGLRE), High Gray Level Run Emphasis (HGLRE), Short Run Low Gray Level Emphasis (SRLGLE), Short Run High Gray Level Emphasis(SRHGLE), Long Run Low Gray Level Emphasis (LRLGLE), Long Run High Gray Level Emphasis (LRHGLE); ***GLGCM*** (Gray Level-GradientCo occurrence Matrix): Small grads dominance, Big grads dominance, Gray asymmetry, Grads asymmetry, Gray mean, Grads mean, Gray variance, Grads variance, Grads Correlation, Gray entropy, Grads entropy, Inertia, Differ moment; applied to Mean and standard deviation, respectively.	Measure the spatial heterogeneity of the image intensity value within the tumor nucleus.
	Intensity	14	Image max, Image mean, Image range, Image median, Image standard deviation, Image variance, Image Mean absolute deviation, Image Skewness, Image Kurtosis, Hist_mean, Hist_variance, Hist_Skewness, Hist_Kurtosis, Hist_Energy	First-order statistical quantification of image intensity characteristics of all tumor nuclei
	Color	54	RGB, HSV, LAB color space histogram statistics including mean, standard deviation, median, variance, range, and max respectively	Color histogram-related statistics of all tumor nuclei
Tumor Core, Invasive Margin	Density	4	Tumor Cell Density, Lymphocyte Density	Ratio of the number of tumor cells and lymphocytes to ROI area
	Uniformity	28	Tumor Cell and Lymphocyte Uniformity matrix: Uniformity mean, Uniformity standard deviation, Uniformity variance, Uniformity median, Uniformity max, Uniformity Range, Uniformity Covariance	Statistical quantification of the distribution of cell numbers in a fixed area of 1024 × 1024 image patch
	Spatial Relation	64	For each tumor cell, calculate the number of tumor cells and lymphocytes within a radius of 50 and 100 pixels: Relation mean, Relation standard deviation, Relation variance, Relation median, Relation max, Relation min, Relation Range, Relation covariance.	Statistical quantification of the distribution of cell numbers across all tumor cells

Reference

1. Lin, T.Y.; Maire, M.; Belongie, S.; Hays, J.; Perona, P.; Ramanan, D.; Dollár, P.; Zitnick, C.L. *Microsoft COCO: Common Objects in Context European Conference on Computer Vision*; Springer International Publishing: Cham, Switzerland, 2014.

© 2020 by the authors. Licensee MDPI, Basel, Switzerland. This article is an open access article distributed under the terms and conditions of the Creative Commons Attribution (CC BY) license (http://creativecommons.org/licenses/by/4.0/).
